# Hidden Markov Model Variants and their Application

**DOI:** 10.1186/1471-2105-7-S2-S14

**Published:** 2006-09-26

**Authors:** Stephen Winters-Hilt

**Affiliations:** 1 Department of Computer Science, University of New Orleans, New Orleans, LA, 70148, USA; 2 The Research Institute for Children, 200 Henry Clay Ave., New Orleans, LA 70118, USA

## Abstract

Markov statistical methods may make it possible to develop an unsupervised learning process that can automatically identify genomic structure in prokaryotes in a comprehensive way. This approach is based on mutual information, probabilistic measures, hidden Markov models, and other purely statistical inputs. This approach also provides a uniquely common ground for comparative prokaryotic genomics. The approach is an on-going effort by its nature, as a multi-pass learning process, where each round is more informed than the last, and thereby allows a shift to the more powerful methods available for supervised learning at each iteration. It is envisaged that this "bootstrap" learning process will also be useful as a knowledge discovery tool. For such an *ab initio *prokaryotic gene-finder to work, however, it needs a mechanism to identify critical motif structure, such as those around the start of coding or start of transcription (and then, hopefully more).

For eukaryotes, even with better start-of-coding identification, parsing of eukaryotic coding regions by the HMM is still limited by the HMM's single gene assumption, as evidenced by the poor performance in alternatively spliced regions. To address these complications an approach is described to expand the states in a eukaryotic gene-predictor HMM, to operate with two layers of DNA parsing. This extension from the single layer gene prediction parse is indicated after preliminary analysis of the *C. elegans *alt-splice statistics. State profiles have made use of a novel hash-interpolating MM (hIMM) method. A new implementation for an HMM-with-Duration is also described, with far-reaching application to gene-structure identification and analysis of channel current blockade data.

## Background

### Motivations for seeking MM and HMM Variants

Part of the problem in developing a reliable gene predictor is having reliable, biologist-verified, training data. In its simplest form, the training data needed is the raw genomic DNA together with a minimal annotation that labels coding regions. For the prokaryotic gene prediction much of the problem with obtaining high-confidence training data can be circumvented by using a bootstrap gene-prediction approach. This is possible in prokaryotes because of their simpler and more compact genomic structure: simpler in that long ORFs (open reading frames) are usually long genes, and compact in that motif searches upstream usually range over hundreds of bases rather than thousands (as in human).

In work on prokaryotic gene prediction (focusing on *V. cholerae*), software (*smORF*) was developed for an augmented ORF (open reading frame) characterization. Using that software, with a simple start-of-coding heuristic, it was possible to establish very good gene prediction for ORFs of length greater than 500 nucleotides. The smORF gene identification was used in a bootstrap gene-annotation process (where no initial training data was provided). The strength of the gene identification was then improved by use of a software tool that performed gap-interpolating-Markov-modeling (gIMM). When applied to the identified coding regions (most of the >500 length ORFs), six gIMMs were used (one for each frame of the codons, with forward and backward read senses). If poorly gIMM-scoring coding regions were rejected, performance improved. Failure analysis clearly indicates that start-codon modeling is needed in order to strengthen predictions. One of the benefits of the gIMM software is its gap-interpolating generalization. This permits motifs to be identified, particularly those sharing the same approximate alignment with the start-of-coding region. Using the bootstrap-identified genes from the smORF-based gene-prediction (including errors) as a train set permitted an unsupervised search for upstream regulatory structure. The classic Shine-Dalgarno sequence (the ribosome binding site) was the strongest signal in the 30-base window upstream from the start codon.

In preliminary work, a Hidden Markov Model based gene predictor was trained and tested on the *C. elegans *genome. Splice signal motifs and intron/exon statistical profiles were extracted, from which a gene predictor was constructed. To boost detection of exons, and indirectly introns, EST information was included. As with the prokaryotic research, further work entails identification of transcription regulation fingerprints, such as the promoter motifs, to clarify starts on transcription, etc. Even with better start-of-coding identification, however, parsing of eukaryotic coding regions by the HMM is still limited by the HMM's single gene assumption, as evidenced by the typically poor gene-prediction performance in alternatively spliced regions. To address these complications an approach is described to expand the states in the gene-predictor HMM to operate with two layers of DNA parsing. This extension from the single layer gene prediction parse was indicated after preliminary analysis of the *C. elegans *alt-splice statistics. State profiles were implemented using a hash-interpolating MM (hIMM) in this process (see Methods).

**Figure 1:**
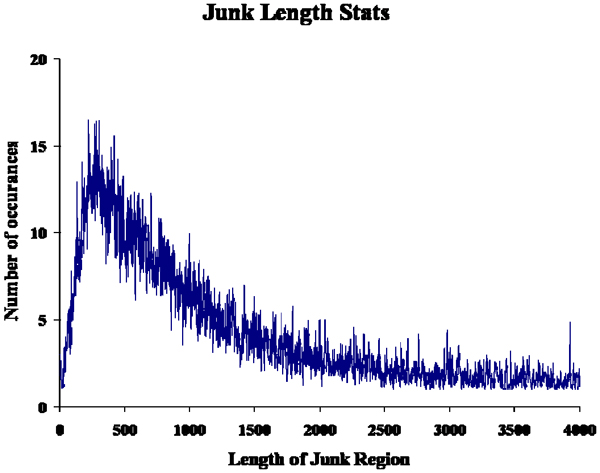
Frequencies of different length junk regions in a test subset of C. elegans.

**Figure 2:**
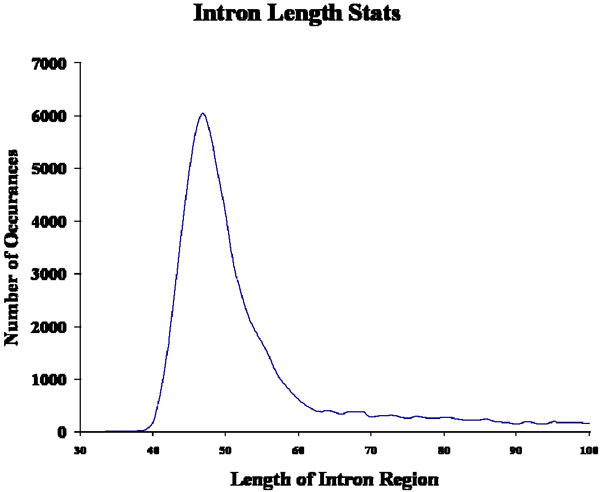
Frequencies of different length intron regions in a test subset of C. elegans (with smoothing).

**Figure 3:**
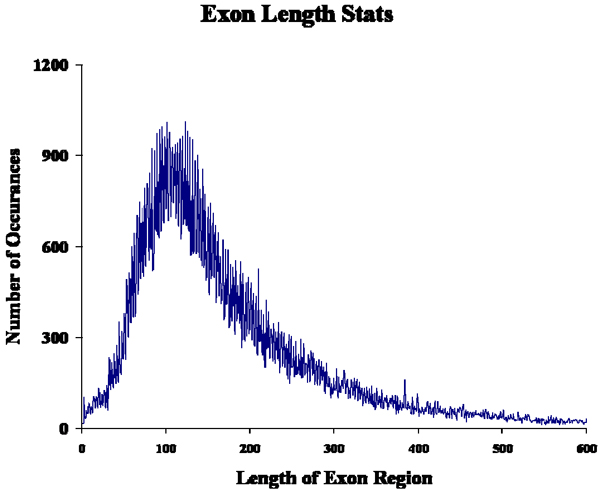
Frequencies of different length exon regions in a test subset of C. elegans.

Another development described here is the incorporation of length distribution information, on exons and introns for example (see Figure 1, figure 2 and figure 3), into the HMM optimization via a novel, algorithmically convenient, implementation of HMM-with-Duration (an HMM that includes true length distribution information). One important application of such an HMM-with-duration includes feature extraction from channel current data. In this setting, information about the mean dwell times for the upper level and lower level is used as a first step in discriminating the class type. HMM with duration incorporates the length distribution information (dwell times distribution) on upper level and lower level states into the HMM optimization.

### Markov Chains

Key property of a Markov chain:

P(x_i _| x_i-1_, ..., x_1_) = P (x_i _| x_i-1_) = ,

where  are sometimes referred to as "transition probabilities" due to their sequential product contribution to sequence probability:

P(x) = P(x_L_, x_L-1 _..., x_1_) = P(x_1_) ∏_i = 2..L _

C_y _is the count of events y, and C_xy _is the count of simultaneous events x and y, T_y _is the count of strings of length one, and T_xy _is the count of strings of length two:

 = P(x | y) = P(x,y)/P(y) = [C_xy_/T_xy_]/[C_y_/T_y_]

Since T_xy_+1 = T_y _→ T_xy _≅ T_y _(sequential data sample property if one long training block),  = C_xy_/C_y _= C_xy_/Σ_x_C_xy_, so C_xy _is complete info for determining transition probabilities.

### Viterbi Path

The recursive algorithm for the most likely state path given an observed sequence (the Viterbi algorithm) is expressed in terms of v_ki_, the probability of the most probable path that ends with observation Z_i _= z_i_, and state S_i _= k. The recursive relation is v_ki _= max_n_{e_ki_a_nk_v_n(i-1)_}, where the max_n_{...} operation returns the maximum value of the argument over different values of index n, and the boundary condition on the recursion is v_k0 _= e_k0_p_k_. The a_kl _are the transition probabilities P(X_i _= l|X_i-1 _= k) to go from state k to state l. The e_kb _are the emission probabilities P(S_i _= b|X_i _= k) while in state k. The Viterbi path labelings are then recursively defined by p(S_i_|S_(i+1) _= n) = argmax_k_{v_ki_a_kn_}, where the argmax_n_{...} operation returns the index n with maximum value of the argument. The evaluation of sequence probability (and its Viterbi labeling) take the emission and transition probabilities as a given. Estimates on those emission and transition probabilities are usually obtained by the Expectation/Maximization (EM) algorithm (commonly referred to as the Baum-Welch algorithm in the context of HMMs [1]).

Further details on the information measures used, such as mutual information, and on Expectation/Maximization (EM), are placed in Appendix A.

### EVA Projection

The HMM method is based on a stationary set of emission and transition probabilities. Emission broadening, via amplification of the emission state variances, is a filtering heuristic that leads to level-projection that strongly preserves transition times between major levels (see [13] for further details). Results from the emission variance amplification (EVA) emission broadening method are described in [13] (with varying amounts of variance amplification). This approach does not require the user to define the number of levels (classes). This is a major advantage compared to existing tools that require the user to determine the levels (classes) and perform a state projection. This allows kinetic features to be extracted with a "simple" FSA (Finite State Automaton) that requires minimal tuning.

## Results

### *Ab initio *prokaryotic gene-finding

**Figure 4:**
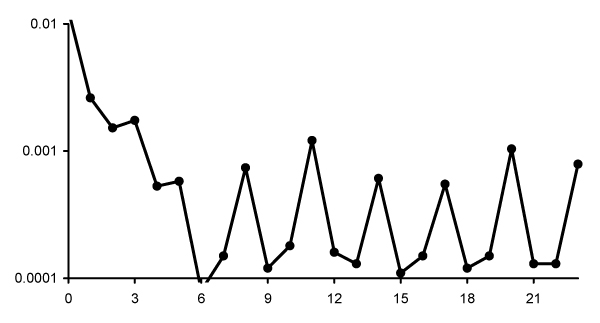
The y-axis shows the mutual information on base-pairs in he *V. cholerae *gemone, the x-axis is the gap size between bases used to construct the base pairs. Mutual Information, MI(X;Y) is: μ = Σ_x_Σ_y_p(xy)log(p(xy)/p(x)p(y)). If X and Y are independent r.v's then MI = 0. If we have a DNA sequence x_1_.... x_i_x_i_+1 x_i+2_........x_n _(where x_k _= {a,c,g, or t}) then we can get counts on pairs x_i_x_i+1 _for i = 1..n, and assuming stationarity on the data, and large enough n, we can speak of the joint probability p(X,Y). Calculation of MI(X,Y) then gives an indication of the linkage between base probabilities in dinucleotide probabilities. This can be extended to linkages when the two bases aren't sequential (have a base gap between them greater than zero), such as pairs based on xixi+2 (gap = 1), etc. This type of statistical framework can then be iterated to higher order MI calculations in a variety of ways to explore a number of statistical linkages and build towards a motif identifier based on such linkages (gIMM). Such an analysis on the *V. Cholerae *Chr. I genome, above, clearly indicates a three-component encoding of data, i.e., a 3-element codon structure is revealed. Furthermore, judging from the strong linkages for gaps 1 through 5, it is also clear that hexamer Markov model statistics will be strong in many regions of the genome.

*Ab initio *gene-finding begins with identification of codon structure on the basis of a simple, genome-wide, mutual information analysis. The idea is to examine the genome's two-base pairings, so first consider pairings where the bases are adjacent in the genome, i.e., a sample set consisting of all dinucleotides found upon moving a window of two-base width across the genome. The counts on those pairings can be used to obtain a mutual information between the first base and second base. The next iteration is to take two-base-pairings separated by one base (gap = 1), etc., with mutual information results as shown in Figure 4 (applied to *V. Cholerae*). This overall method is generalized further to define a gap-interpolating Markov model (details in Methods).

**Figure 5:**
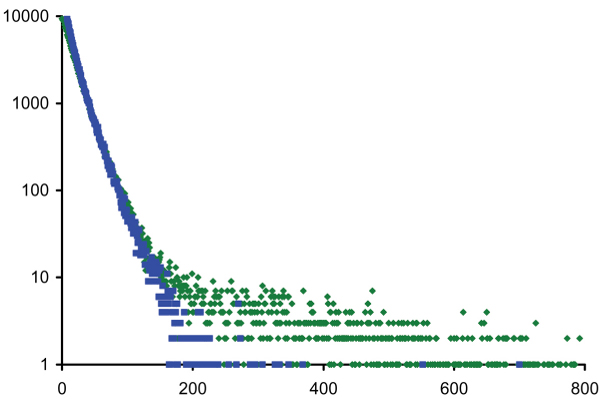
An examination of the void sizes encountered for various codons (smORFs), or groupings of codons, reveals the stop codons very clearly as codons with anomalously lengthy stop-codon void regions. Shown in green are the standard ORFs (voids in the codon subset {(taa),(tag),(tga)}), which are clearly behaving differently from other codon voids (blue) when length is greater than 500 bases. The voids shown in blue are voids in the codon subset {(aaa),(gaa),(gat)}.

**Figure 6:**
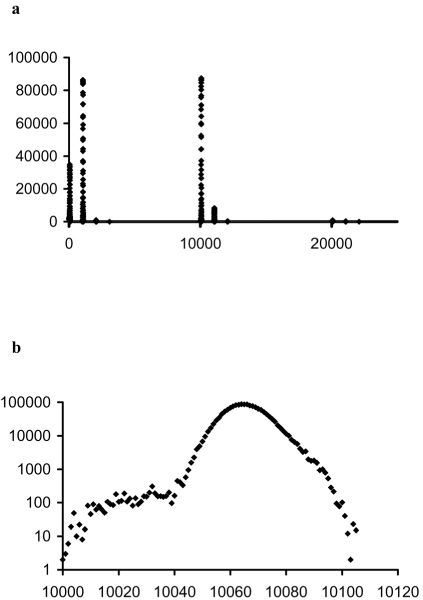
**a**. Topology Index Histograms shown for the *V. cholerae *Chr. I genome, where the x-axes are the topology index, and the y-axes show the event counts (i.e., occurance of that particular topolgy index in the genome). The topology index is computed by the following scheme: (i) initialize index for all bases in sequesnce to zero. (ii) Each base in a forward sense ORF, with length greater than a specified cut-off, is incremented by +10,000 for each such ORF overlap. Similarly, bases in reverse sense ORFs are incremented by +1,000 for each such overlap. Voids larger than the cut-off length in the non-standard smORFs each give rise to an increment of +1. The figure shows that *V. cholerae *only has a small portion of its genome involved in multiple gene encodings. **b**. Shows a close-up view of the 10000 topology index peak.

**Figure 7:**
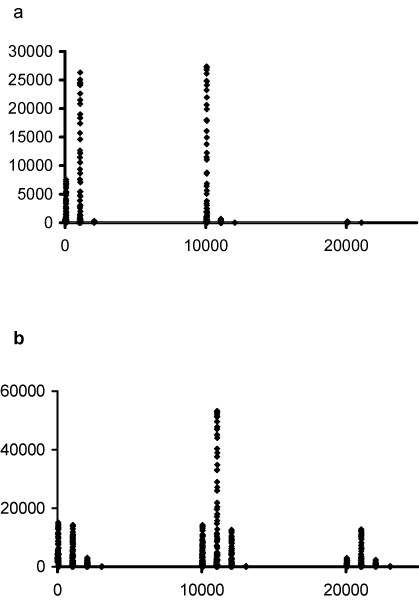
**a**. Topology-Index histograms are shown for the *Chlamydia trachomatis *genome. **b**. Topology-Index histograms are shown for the *Deinococcus radiodurans *genome. *C. trachomati*s, like *V. cholerae*, shows very little overlapping gene structure. *D. radiodurans*, on the other hand, is dominated by genes that overlap other genes (note the strong 11000 peak).

*Ab initio *gene-finding can then identify the stop codons and, thus, ORFs (see Figure 5). A generalization to codon void regions, then, leads to recognition of different, overlapping, potential gene regions (with two orientations). A tool has been developed to identify the genomic ORF topology in this generalized way, named "*smORF*" (see Figure 6 and Table 1 for further definitions and results). This genome-topology tool also clearly shows differences between bacteria (a possible "fingerprint" tool) (see Figure 6 and Figure 7).

*smORF *offers information about open reading frames (ORFs), and tallies information about other such codon void regions (an ORF is a void in three codons: TAA, TAG, TGA). This allows for a more informed selection process when sampling from a genome, such that non-overlapping gene starts can be cleanly and unambiguously sampled. The goal is, initially, to identify key gene structures (e.g., stop codons, etc.) and use only the highest confidence examples to train profilers. Once this is done, Markov models (MMs) can be constructed on the suspected start/stop regions and coding/noncoding regions. The algorithm then iterates again, informed with the MM information, and partly relaxes the high fidelity sampling restrictions (essentially, the minimum allowed ORF length is made smaller). A crude gene-finder can be constructed on the high fidelity ORFs by use of a very simple heuristic: scan from the start of an ORF and stop at the first in-frame "atg". This analysis was applied to the *V. cholerae *genome (Chr. I). 1253 high fidelity ORFs were identified out of 2775 known genes. This first-"atg" heuristic provided a gene prediction accuracy of 1154/1253 (92.1% of predictions of gene regions were exactly correct). If small shifts are allowed in the predicted position of the start-codon relative to the first-"atg" (within 25 bases on either side), then prediction accuracy improves to 1250/1253 (99.8%). This actually elucidates a key piece of information needed to improve such a prokaryotic gene-finder. Basically, information is needed to help identify the correct start codon in a 50 base window. Such information exists in the form of DNA motifs corresponding to the binding footprint of regulatory biomolecules (that play a role in transcriptional or translational control).

For an *ab initio *gene-finder to work, it will need to have a mechanism to identify critical motif structure, such as those around the start of coding or start of transcription (and then, hopefully more). In essence, a Markov model is needed with greater "reach" – the gap-interpolating Markov model (gIMM) was developed for this purpose, and is described in the Methods. To set up an *ab initio *motif discovery windows around the (1253) purported start of genes were sampled. The windows ranged from the 40 bases preceding the start codon to the first 20 bases of coding (a 60 base window). Some of the windows represent noise, as the first pass of the bootstrap feature extraction has only 92.1% accuracy. Even so, the gIMM is able to clearly discern the Shine-Dalgarno consensus sequence. With the critical motifs already discerned, further iterations of the MM construction, possibly as an HMM now, will undoubtedly aid in improving performance.

### Alternate-Splice Labeling Scheme for Eukaryotic HMM

The labeling scheme assigns a label to each base in the sequence. Exon frame position 0 bases have label 0 if in the forward read or A if in the reverse. Likewise, exon frame position 1 bases have label 1 or B, and exon frame position 2 bases have label 2 or C. Introns, for purposes of the analysis here, are represented as 'i' or 'I' for intron on the forward or reverse strand (in the HMM implementation the intron states are actually split out in order to maintain proper frame on re-entry to coding regions via state transition restrictions). Junk DNA is labeled 'j'.

#### Track Label Information

Suppose there were multiple annotations regarding the labeling of a base (i.e., alternative splicing). As the genome is traversed in the forward direction, gene annotations that aren't in conflict with annotations already seen are used to determine labels on label-track-one. If a gene annotation is in conflict (an alternative splicing) then its label information is recorded on a second, adjacent, label track. Table 2 shows the label counts on track one and on track two (where the default base label is taken to be 'j'). From Table 2 it can be seen that about 8% of the first chromosome of *C. elegans *genes has alternate splicing. Similarly, Table 3 shows the transition counts between labels.

#### V-Labels and V-Transitions

Counts on coding-overlap V-label are shown in Table 4. Notice how the V-labels tend NOT to favor overlapping that is a simple frame-shift in a given read direction (i.e., the V01 count is very low compared to V00, etc.). There are 263 transitions on V-labels with non-zero counts. Many of the V-transitions have very low counts and can either be ignored in the initial model, or can have their stat's boosted by bringing information from related genomes (*C. Briggsae*). Ignoring those V-transitions with negligible counts as not allowed transitions, as well as those implicitly describing no alternative splicing locally (an overlap with 'j' in either track), reduces to an active V-transition set consisting of 86 transitions between V-labels. This is a tractable number of states to manage in the HMM analysis, suggesting a simple and direct approach to alternative splice HMM analysis. The number of V-transitions, whether counting all 263, or the 86 'active' ones, is still much smaller than the 72*72 = 5,184 transitions that would have been surmised for track annotations that were entirely independent.

### HMM-with-Duration with application to channel current signal analysis

#### Synthetic Data Generation

**Figure 8:**
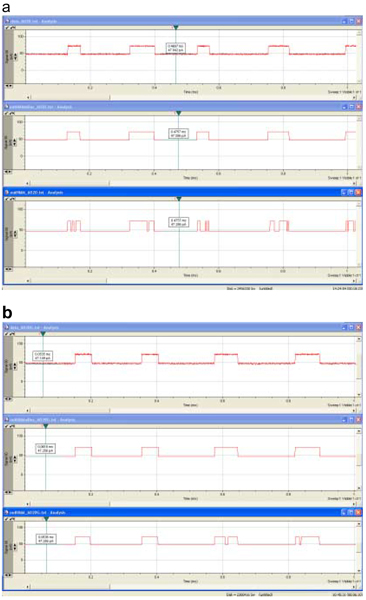
**a**. Synthetic data with Poisson distributed length statistics is shown in the upper trace. Emission broadening is introduced with an emission variance amplification factor of 20. This effectively broadens the noise band (thickness) seen in the upper trace by a factor of 20, which leads to a blurring between the upper and lower levels of blockade since the noise bands now overlap (i.e., a toggling cross-over instability is introduced to challenge the projection method). The middle trace shows the clean, highly accurate Viterbi parsing into the appropriate levels that is still obtained with the HMM-with-Duration implementation. The lower trace shows the Viterbi parse with a simple HMM, that is uninformed about the underlying length distributions, thus giving rise to a Viterbi traceback parse that fails to penalize unlikely, very short duration, blockade events (seen as the unstable, rapid level-projection toggles). **b**. Synthetic data with Gaussian distributed length statistics is shown in the upper trace, with the suceessful HMM-with-Duration parsing shown in the middle trace. Emission broadening is introduced with an emission variance amplification factor of 20 as in Fig. 8a, with a similar failure in the HMM-*without*-Duration's ability to parse critical kinetic feature information.

We have generated two sets of synthetic data with states upper level (UL) and lower level (LL) (see Figure 8). The life times of the UL & LL are drawn from a Poisson distribution. The means of UL & LL dwell times for data set 1 are 20 ms & 40 ms respectively. The means of UL & LL dwell times for data set 2 are 40 ms each. A mean value of 48 pA is used for LL and a mean value of 72 pA is used for UL. We have assumed that the noise at UL & LL vary at around +/- 5pA around their respective means (Gaussian distribution). Hence we have assumed a variance of 2.78. See Figure 8.

The HMM-with-Duration implementation (described in the Methods) has been tested in terms of its performance at parsing synthetic blockade signals with attributes and visual appearance almost indiscernible from the experimentally observed blockade data. The benefit of the synthetic data is that the mean lifetimes of the blockade levels can range over an exhaustive set of possibilities for thorough testing of the HMM-with-Duration. The synthetic data was designed to have two levels, with lifetime in each level determined by a governing distribution (Poisson and Gaussian distributions with a range of mean values were considered). In Figure 8, the data was generated with Poisson distributed lifetimes and parsed by an HMM's with and without duration, with length distribution generated from a Poisson (Figure 8a) or a Gaussian (Figure 8b) Distributions. The results clearly demonstrate the superior performance of the HMM-with-duration over its simpler, HMM without Duration, formulation. With use of the EVA-projection method described in the Background (and in [13]) this affords a robust means to obtain kinetic feature extraction. The emission broadening introduced in the HMM w/wo duration comparison in Figure 8a and 8b, is precisely what occurs with EVA-projection, with similar susceptibility to failure due to over-representation of brief blockade lifetimes in the HMM without Duration parse. Using HMM with duration this problem is resolved, brief toggles between states are now penalized according to the statistical rarity of the comparatively brief lifetime events (see Fig.'s 8a and 8b). Resolving this problem is critical for accurate kinetic feature extraction, and the results suggests that this problem can be elegantly solved with a pairing of the HMM-with-Duration stabilization with EVA-projection.

## Discussion

### aTOPO

As already mentioned, the gIMM tool identifies statistically anomalous motifs (usually restricted to some zone upstream). Another tool being developed, "aTOPO" (for motifs with Anomalous TOPOlogy), uses the information from the upstream zones swept with the gIMM tool to annotate upstream motifs, and offers further information on whether an anomalous motif is biologically significant by looking at is positional distribution and its correlations to other motifs. This work is still preliminary, so this will not be discussed further.

## Methods

### Expectation/Maximization

Expectation/Maximization, EM, is a general method to estimate the maximum likelihood when there is hidden or missing data. The method is guaranteed to find a maximum, but it may only be a local maximum, as is shown here (along the lines of [1]). For a statistical model with parameters θ, observed quantities x, and hidden labels π, the EM goal is to maximize the log likelihood of the observed quantities with respect to θ : log P(x|θ) = log[Σ_π_P(x,π|θ)]. At each iteration of the estimation process we would like the new log likelihood, P(x|θ), to be greater than the old, P(x|θ*). The difference in log likelihoods can be written such that one part is a relative entropy, the positivity of which makes the EM algorithm work:

log P(x|θ) - log P(x|θ*) = Q(θ|θ*) - Q(θ*|θ*) + D[P(π|x,θ*)||P(π|x,θ)],

where D[...||...] is the Kullback-Leibler divergence, or relative entropy, and Q(θ|θ*) = Σ_π_P(x,π|θ). Now a greater log likelihood results simply by maximizing Q(θ|θ*) with respect to parameters θ. The EM iteration is comprised of two steps: (1) Estimation – calculate Q(θ|θ*), and (2) Maximization – maximize Q(θ|θ*) with respect to parameters θ.

For an HMM the hidden labels π correspond to a path of states. Along path π the emission and transition parameters will be used to varying degrees. Along path π, denote usage counts on transition probability a_kl _by A_kl_(π) and those on emission probabilities e_kb _by E_k_(b,π) (following [1] conventions), P(x,π|θ) can then be written:

P(x,π|θ) = ∏_k = 0_∏_b_[e_kb_]^E_k_(b,π) ∏_k = 0_∏_l = 1_[a_kl_]^A_kl_(π).

Using the above form for P(x,π|θ), A_kl _for the expected value of A_kl_(π) on path π, and E_k_(b) for the expected value of E_k_(b,π) on path π, it is then possible to write Q(θ|θ*) as:

Q(θ|θ*) = Σ_k = 1_Σ_b _E_k_(b) log[e_kb_] + Σ_k = 0_Σ_l = 1 _A_kl _log[a_kl_].

It then follows (relative entropy positivity argument again) that the maximum likelihood estimators (MLEs) for a_kl _and e_kb _are:

a_kl _= A_kl _/(Σ_l _A_kl_) and e_kb _= E_k_(b)/(Σ_b _E_k_(b)).

The latter estimation is for when the state sequence is known. For an HMM (with Baum-Welch algorithm) it completes the Q maximization step (M-step), which is obtained with the MLEs for a_kl _and e_kb_. The E-step requires that Q be calculated, for the HMM this requires that A_kl _and E_k_(b) be calculated. This calculation is done using the forward/backward formalism with rescaling in the next section.

### Emission and Transition Expectations with Rescaling

For an HMM, the probability that transition a_kl _is used at position i in sequence Z is:

p(S_i _= k, S_(i+1) _= l|X) = p(S_i _= k, S_(i+1) _= l, Z)/p(Z), where

p(S_i _= k,S_(i+1) _= l,Z) = p(z_0_,...,z_i_,S_i _= k)p(S_(i+1) _= l|S_i _= k)p(z_i+1_|S_(i+1) _= l)p(z_i+2_,...,z_L-1_|S_(i+1) _= l).

In terms of the previous notation with forward/backward variables:

p(S_i _= k, S_(i+1) _= l|X) = f_ki _a_kl _e_l(i+1) _b_l(i+1) _/p(Z),

So the expected number of times a_kl _is used, A_kl_, simply sums over all positions i (except last with indexing):

A_kl _= Σ_i _f_ki _a_kl _e_l(i+1)_b_l(i+1)_/p(Z),

Similarly, the probability that b is emitted by state k at position i in sequence Z:

p(z_i _= b, S_i _= k|X) = [ p(z_0_,...,z_i_,S_i _= k) p(z_i+1_,...,z_L-1_|S_i _= k)/p(Z) ] δ(z_i_-b),

where a Kronecker delta function is used to enforce emission of b at position i. The expected number of times b is emitted by state k for sequence Z:

E_k_(b) = Σ_i _f_ki _b_ki_/p(Z) δ(z_i_-b),

In practice, direct computation of the forward and backward variables can run into underflow errors. Rescaling variables at each step can control this problem. One rescaling approach is to rescale the forward variables such that Σ_i_F_ki _= 1, where F_ki _is the rescaled forward variable, and B_ki _is the rescaled backward variable: F_ki _= a_βk_e_ki_F_β(i-1)_/s_i_, and B_ki _= a_kβ_e_β(i+1) _B_β(i+1)_/t_i+1_, where s_i _and t_i+1_, are the rescaling constants. The expectation on counts for the various emissions and transitions then reduce to:

A_kl _= Σ_i _F_ki _a_kl _e_l(i+1) _B_l(i+1)_/[Σ_k_Σ_l _F_ki _a_kl _e_l(i+1)_] and E_k_(b) = Σ_i _F_ki _B_ki _δ(z_i_-b).

### Gap Interpolating Markov Model (gIMM)

We construct a gIMM using Mutual Information (MI). Based on the three reading frames of the DNA, the Mutual Information is frame dependent.

 Find out which position among the 24 prior positions has the maximum mutual information with frame position 0. Suppose it is p1

Frame positions: ...012012012012012012012|012012...

Prior positions: ...------------------987654321

 Find out a position from p = 1...24 and p ≠ p1 such that it has the maximum mutual information with frame position 0 and the prior position p1. If it is p2: MI(x0,x1;x2)>MI(x0,x1;xk).

 Repeat the above procedure until we get an nth-order MI-linkage, this defines an interpolated Markov model that allows for gaps (gIMM). Likewise, for the frame 1 and 2, and reverse frames.

 Most importantly, this can also be done for the regions upstream of coding regions, with reference base just prior to the start codon, in searches for regulatory motifs.

### Hash Interpolated MM (hIMM)

 Given the current state and the emitted sequence as x1,...., xL; compute:

P(xL|x1,...., xL-1) ≈ Count(x1,...., xL)/Count(x1,...., xL-1)

Iff Count(x1,...., xL-1) ≥ 400 i.e. only if the parental sequence shows statistical significance

 Store P(xL|x1,...., xL-1) in the hash

 Maintain a separate hash for each of the following states – Junk, Intron and Exon0, Exon1 & Exon2

Pseudo Code:

 The emission probabilities for states such as (jj), (ee) and (ii) are computed using hash IMM as:

Given sequence 'x1,...., xL'

If sequence is defined;

Return Probability

Else

Recurse on 'x1,...., xL-1'

 Minimum string length allowed for:

1. Junk Region = 6

2. Intron Region = 6

3. Exon Region = 8

### UL/LL Data Generation Method

We generated 75000 random numbers that were Gaussian distributed with mean 48 pA and variance 2.78. We also generated 50000 random numbers that were Gaussian distributed with mean 72 pA and variance 2.78.

Since the dwell times of the UL & LL are Poisson distributed, the transition times (duration times between state transitions) are assumed to be exponentially distributed. Therefore for data set 1, the mean interval between successive UL states is 40 ms and the mean interval between successive LL states is 20 ms.

Cumulant Distribution Function (CDF) of exponential distribution:

F = 1 - exp (-t/a) where 'a' is the mean

Hence t = a.log_e_(1 - F)

We generate uniformly distributed 'F' between [0, 1). We then compute 't' from the above equation.

We have 50,000 samples per second. Hence a dwell time of 't' ms corresponds to 50.t samples rounded off to the nearest integer.

### HMM-with-Duration via Cumulant Transition Probabilities

 The transition probabilities for (ee) – (ee); (jj) – (jj) and (ii) – (ii) type transitions are computed as:

Prob(jj|j_length _= L) = Prob(j_length _≥ L+1)/Prob(j_length _≥ L)

 The transition probabilities for transitions such as (jj) – (je), (ee) – (ej), (ee) – (ei), (ii) – (ie) are computed as:

If the total number of (ej) transitions is 60 and the total number of (ei) transitions is 40, then:

Prob(ei|e_length _= L) = Prob(e_length _= L)/Prob(e_length _≥ L) × 40/(40 + 60)

Prob(ej|e_length _= L) = Prob(e_length _= L)/Prob(e_length _≥ L) × 60/(40 + 60)

Pseudo Code:

 Maintain separate counters for the junk, exon and intron regions.

 The counters are updated as:

1. The exon counter is set to 2 for a (je) – (ee) transition

2. The exon counter gets incremented by 1 for every (ee) – (ee) transition

 Prob(e_length _≥ L+1) is computed as:

We have Prob(e_length _≥ L+1) = 1 - Σ_i = 1..L _Prob(e_length _= i)

Hence we generate a list such that for each index 'k > 0', the value 1 - Σ_i = 1..k _Prob(e_length _= i) is stored

### HMM-with-Duration Viterbi Implementation

 The transition probabilities for (UL) – (UL); (LL) – (LL) are computed as:

Prob(ul|ul_len = L) = Prob(ul_len ≥ L+1)/Prob(ul_len ≥ L)

 The transition probabilities for transitions such as (UL) – (LL), (LL) – (UL) are computed as:

Prob(ul-ll|ul_len = L) = Prob(ul_len = L)/Prob(ul_len ≥ L)

**Figure 9:**
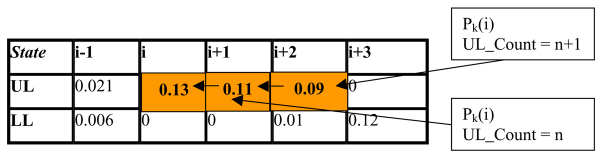
The HMM dynamic programming table is modified to track new count index information at each cell to enable the HMM-with-Duration implementation. The figure shows a view of Viterbi modifications in the dynamic programming table – the transition probabilities are now modified to be a ratio of transition probability cumulants. The transition probability cumulant is calculated based on a predetermined length distribution profile and uses information on the length that is also a (new) cell-level parameter. There are two new cell-level parameters, one each for tracking length on UL and LL states. (For gene-finding, the information to track at the cell-level would be the length of the purported exon, intron, or junk, region.)

Pseudo Code (see dynamic programming table in Figure 9):

 Maintain separate counters for the UL and LL regions

 The counters are updated as:

1. The UL counter is set to 1 for a (ll) – (ul) transition

2. The UL counter gets incremented by 1 for every (ul) – (ul) transition

 Prob(ul_len ≥ L+1) is computed as:

We have Prob(ul_len ≥ L+1) = 1 - Σ_i = 1 _^L ^Prob(ul_len = i)

Hence we generate a list such that for each index 'k > 0', the value 1 - Σ_i = 1 _^k^Prob(ul_len = i) is stored

 Prob(ul_len = L) is computed as:

We generate a list such that for each index 'k', the value Prob(ul_len = k) is stored

## Appendix A

### Information measures

The fundamental information measures are Shannon entropy, mutual information, and relative entropy (also known as the Kullback-Leibler divergence or distance). Shannon entropy: σ = -Σ_x_p(x)log(p(x)), is a measure of the information in distribution p(x). Mutual Information: μ = Σ_x_Σ_y_p(xy)log(p(xy)/p(x)p(y)), is a measure of information one random variable has about another random variable. Relative Entropy (Kullback-Leibler distance): ρ = Σ_x _p(x) log(p(x)/q(x)), is a measure of distance between two probability distributions. Mutual information is a special case of relative entropy between a joint probability (two-component in simplest form) and the product of component probabilities.

### Hidden Markov Models with possible Side Information

Hidden Markov Models [1] provide a statistical framework for sequences of observations obeying stationary Markov statistics. The "hidden" part of the HMM consists of the labelings, s_i_, for each observation, z_i_, where the index i labels the observation. The stationary statistics for a first order HMM are described in terms of emission probabilities, e_ni _= p(Z_j _= z_i_|S_j _= n), and transition probabilities, a_nm _= p(S_j _= m|S_(j-1) _= n). (The indexing on j is left in for clarity on the transition probability definition; from stationarity the expressions are valid for any choice of j.) Given (i) sequence of observations z_i_, (ii) hidden labels s_i_, and (iii) stationary Markov statistics, one can calculate: (i) p(Z_0...L-1_), or (ii) the most likely hidden labeling (path with largest contribution to p(Z_0...L-1_)), or (iii) the re-estimation of emission and transmission probabilities such that p(Z_0...L-1_) is maximized (using Expectation/Maximization).

### Forward and Backward Variables

The forward/backward variables can be used to evaluate p(Z_0...L-1_) by breaking the sequence probability p(Z_0...L-1_) into two pieces via use of a single hidden variable treated as a Bayesian parameter: p(Z_0...L-1_) = Σ_k_p(Z_0...i_,s_i _= k)p(Z_i+1...L-1_,s_i _= k) = Σ_k_f_ki_b_ki_, where f_ki _= p(Z_0...i_,s_i _= k) and b_ki _= p(Z_i+1...L-1_,s_i _= k). A proof for the two-piece split is obtained by directly expanding the sequence probability (via Chow expansion: P(xyz) = P(x)P(y|x)P(z|xy), and Markov conditional reduction: P(xyz) = P(x)P(y|x)P(z|y), with appropriate notational book-keeping). Given stationarity, the state transition probabilities and the state probabilities at the i^th ^observation satisfy the trivial relation p_qi _= Σ_k_a_kq_p_k(i-1)_, where p_qi _= p(S_i _= q), and p_q0 _= p(S = q), and the latter probabilities are the state priors. The trivial recursion relation that is implied can be thought of as an operator equation, with operation the product by a_kq _followed by summation (contraction) on the k index. The operator equation can be rewritten using an implied summation convention on repeated Greek-font indices (Einstein summation convention): p_q _= a_βq_p_β _. Transition-probabilities in a similar operator role, but now taking into consideration local sequence information via the emission probabilities, are found in recursively defined expressions for the forward variables, f_ki _= a_βk_e_ki _f_β(i-1)_, and backward variables, b_ki _= a_kβ_e_β(i+1) _b_β(i+1)_. The recursive definitions on forward and backward variables permit efficient computation of observed sequence probabilities using dynamic programming tables. It is at this critical juncture that side information must mesh well with the states (column components in the table), i.e., in a manner like the emission or transition probabilities. Length information, for example, can be incorporated via length-distribution-biased transition probabilities (as described in a new method in the Methods).

## Figures and Tables

**Table 1:** Topology-Index Histogram results are shown for *Vibrio Cholerae*. The N = 400 set correspond to indexing with ORFs greater than 400 bases long. Similarly, the N = 200 set correspond to indexing with ORFs greater than 200 bases long.

**N = 400**		**N = 200**	
Score	Count	Score	Count

0	1653287	0	846889
1000	649241	1000	1017629
2000	400	2000	1554
10000	630232	10000	954394
11000	27986	11000	138817
20000	3	12000	161
		20000	1700
		21000	5

**Table 2:** (a) shows the Track 1 Label Counts, and (b) shows the Track 2 Label Counts.

(a)		
0 : 571,187	A : 518,431	I : 1,634,653
1 : 571,187	B : 518,431	i : 1,779,392
2 : 571,187	C : 518,431	j : 7,336,733
(b)		
0 : 21,599	A : 64,475	I : 325,471
1 : 21,599	B : 64,471	i : 81,289
2 : 21,599	C : 64,467	j : 13,354,661

**Table 3:** (a) shows the Track 1 Transition Counts, and (b) shows the Track 2 Transition Counts.

(a)		
01 : 569,483	BA : 516,874	II : 1,628,572
12 : 569,490	CB : 516,868	ii : 1,772,795
20 : 566,732	AC : 514,309	jj : 7,334,177
0i, i1 : 1,704	IA, BI : 1,557	j0, 2j : 1,257
1i, i2 : 1,696	IB, CI : 1,563	Aj, jC : 1,161
2i, i0 : 3,197	IC, AI : 2,961	
(b)		
01 : 21,554	BA : 64,296	II : 324,751
12 : 21,548	CB : 64,275	ii : 81,073
20 : 21,441	AC : 63,986	jj : 13,354,350
0i, i1 : 45	IA BI : 175	j0, 2j : 38
1i, i2 : 51	IB, CI : 192	Aj, jC : 136
2i, i0 : 120	IC, AI : 353	

**Table 4:** V-label Counts.

V00, V11, V22: 17,839	VA0, VB2, VC1: 0
V01, V12, V20: 3	VA1, VB0, VC2: 0
V02, V10, V21: 58	VA2, VB1, VC0: 829
V0A, V1C, V2B: 741	VAA, VBB, VCC: 16,169
V0B, V1A, V2C: 957	VAB, VBC, VCA: 0
V0C, V1B, V2A: 5164	VAC, VBA, VCB: 54
